# Non-Destructive Imaging and Spectroscopic Techniques for Assessment of Carcass and Meat Quality in Sheep and Goats: A Review

**DOI:** 10.3390/foods9081074

**Published:** 2020-08-07

**Authors:** Severiano Silva, Cristina Guedes, Sandra Rodrigues, Alfredo Teixeira

**Affiliations:** 1 Veterinary and Animal Research Centre (CECAV) Universidade Trás-os-Montes e Alto Douro, Quinta de Prados, 5000-801 Vila Real, Portugal; cguedes@utad.pt; 2 Mountain Research Centre (CIMO), Escola Superior Agrária/Instituto Politécnico de Bragança, Campus Sta Apolónia Apt 1172, 5301-855 Bragança, Portugal; srodrigues@ipb.pt (S.R.); teixeira@ipb.pt (A.T.)

**Keywords:** sheep, goats, carcass, meat, imaging techniques, spectroscopic techniques

## Abstract

In the last decade, there has been a significant development in rapid, non-destructive and non-invasive techniques to evaluate carcass composition and meat quality of meat species. This article aims to review the recent technological advances of non-destructive and non-invasive techniques to provide objective data to evaluate carcass composition and quality traits of sheep and goat meat. We highlight imaging and spectroscopy techniques and practical aspects, such as accuracy, reliability, cost, portability, speed and ease of use. For the imaging techniques, recent improvements in the use of dual-energy X-ray absorptiometry, computed tomography and magnetic resonance imaging to assess sheep and goat carcass and meat quality will be addressed. Optical technologies are gaining importance for monitoring and evaluating the quality and safety of carcasses and meat and, among them, those that deserve more attention are visible and infrared reflectance spectroscopy, hyperspectral imagery and Raman spectroscopy. In this work, advances in research involving these techniques in their application to sheep and goats are presented and discussed. In recent years, there has been substantial investment and research in fast, non-destructive and easy-to-use technology to raise the standards of quality and food safety in all stages of sheep and goat meat production.

## 1. Introduction

The rapid assessment of carcass composition and meat quality is valuable for the development of breeding programs oriented to the market and for a value-based payment and marketing system [1,2] and also to achieve the assumption of farm animal production in the near future as resilient, adjusted and producing safe and healthy food [3,4,5]. To achieve those ongoing challenges, much research has been conducted to develop rapid, non-destructive and non-invasive techniques to apply to animal production and the meat industry. In the last decade, a number of improvements have been made to the quantitative assessment of carcass and meat traits using imaging and spectroscopic techniques [6,7], along with tools for assessing and analysing images and new algorithms to effectively predict carcass and meat quality traits in the industry [8,9,10]. For all meat species, comprehensive studies using imaging and spectroscopic techniques to assess carcass and meat traits have recently been published [7,11]. However, there is a gap in the knowledge of these approaches for sheep and goat species. It is, therefore, the objective of this work to summarize the most recent developments in the use of imaging and spectroscopic techniques applied to sheep and goats.

## 2. Imaging Techniques to Assess Carcass and Meat Quality in Sheep and Goats

In the last decade, significant development of non-invasive and non-destructive imaging techniques to obtain objective data in carcass and meat quality has been made. In this section, recent improvements on the use of dual-energy X-ray absorptiometry (DXA), computed tomography (CT) and magnetic resonance imaging (MRI) to assess sheep and goat carcass and meat quality will be discussed.

### 2.1. Dual-Energy X-Ray Absorptiometry

Dual-energy X-ray absorptiometry (DXA) is a widely accepted method for the assessment of body composition in human medicine/research [12,13] and also in meat-producing species [14]. Using the Web of Science search functions, it was possible to identify 59 articles related to DXA in pigs (*n* = 44), cattle (*n* = 3), broilers (*n* = 3) and sheep (*n* = 9). No work was found in goats. Table 1 summarizes the main results of research using DXA to predict fat (% and g) and lean (% and g) of sheep carcasses or in vivo.

The studies with DXA have as main objective the determination of body or carcass traits that have importance for the meat industry [14] or animal nutrition [15]. Comparing the results of DXA studies is not easy because some works focus on predicting the composition based on the amount of chemical or dissected tissues rather than by the proportion, and this way the composition is powerfully described by the carcass weight and not by the DXA value. Furthermore, many of the studies were structured around samples of a reduced number of animals and, therefore, tend to report accuracy indicators as results within the sample as opposed to the precision demonstrated across a series of slaughter groups over time [16]. The data in Table 1 show that, despite some modest results, DXA is a technique capable of predicting sheep carcass composition, rather than other carcass characteristics, which has led several authors to consider it as very capable for use in the sheep meat industry [16,17,18]. In a work in which the same equipment was always used (GE Lunar DPX-IQ), it was observed that the accuracy tends to be higher in pigs than in sheep [19]. For in vivo studies, the whole body analysis of sheep is strongly affected by the gastrointestinal tract, resulting in less accuracy compared to pigs [19]. Despite these limitations, the DXA has been used as an alternative to dissection or chemical composition of sheep carcasses using equations developed in previous studies. For example, in an investigation [20] that aimed to study the effect of different finishing diets on carcass traits of lambs, a DXA machine was used that incorporated calibration equations described in a previous study [21], which found high prediction accuracies for lean weight (R^2^ = 0.98) and its percentage (R^2^ = 0.94). Additionally, Hunter et al. [17] stated that the estimates obtained by DXA of the weight of tissues of sheep could be used to predict the in vivo chemical composition of the body (R^2^ between 0.977 and 0.999) or the carcass chemical composition (R^2^ between 0.906 and 0.998). In any case, and despite the high value of accuracy of the prediction models, its application must take into account the need for calibration and aspects such as the type of equipment, species, weight and age of the animals.

Obtaining information on the lamb carcass composition at the chain-speed of a slaughterhouse is very challenging. Still, to address saleable meat yield, which is negatively affected by the excess of fat, it is necessary to obtain information on the carcass composition [16]. To understand the suitability of DXA for use in an abattoir, these authors conducted a study at the chain-speed with 607 lamb carcasses. The carcass reference composition was obtained by CT, which determined the percentages of fat, lean and bone. The results show excellent accuracy of DXA models for fat% estimation (RMSE = 1.32 and R^2^ = 0.89), but less accuracy to predict lean % (RMSE = 1.69 and R^2^ = 0.69) and bone % (RMSE = 0.68 and R^2^ = 0.68).

With the technological advancement of equipment and software, DXA can have the similar accuracy as CT and MRI in the prediction of carcass and body composition [19]. Obtaining 3D measurements with DXA represents a step forward in the use of this technique [19]. There is already equipment that combines DXA and CT technology called DECT (dual-energy computed tomography) that has been used successfully in the medical field [29]. The possibility of using this type of equipment for animal imaging in abattoirs or in vivo performance testing represents an opportunity for animal science [19]. Recently, DXA was validated in a chain-speed abattoir with a diverse lamb carcass sample, both from a genotypic and phenotypic point of view [16,18], and showed that DXA could be a solution that provides confidence to producers about the carcass information. This confidence can be the key to a value-based market system supported by the quantification of saleable meat yield.

### 2.2. Computed Tomography

Computed tomography (CT) is a fundamental tool for medical diagnosis [30]. This technique has undergone significant advances in recent years, with reducing costs of acquisition. Nevertheless, it remains an expensive technique in equipment and operating costs. In animal science, particularly in meat production, CT has been recognized as useful since the beginning in the early 1980s [31]. Despite its high complexity and cost, in the last ten years CT has gained importance for the knowledge of body and carcass composition and meat quality of sheep [14,32]. Much of this knowledge results from research and practical application in several countries with both mobile and fixed CT [33]. For sheep, CT has been identified as an alternative to carcass dissection, which is destructive, time-consuming, costly and inadequate for genetic selection programs [32]. That is why the CT applied in vivo has had a tangible impact on the genetic progress for sheep carcass characteristics [34]. Therefore, the relatively high costs of selection programs based on CT traits needs to be put into perspective to its benefits [35]. Permanent access to information related to CT images and databases of the composition traits must also be considered as valuable [14]. It should also be noted that CT, as a non-invasive technique, allows the modelling of the body composition of the same animal over time, which adds precision to the decisions about breeding and nutrition [36,37]. Table 2 presents a summary of works that use CT for prediction of carcass or body composition of sheep and goats.

Most of the early CT work with sheep uses two-dimensional images of selected anatomical landmarks of the object [45,46]. As a result, the compromise between precision and the number of anatomical landmarks was examined and a methodology with three cross-sectional CT scans was elected [40,45]. With three cross-sectional CT scans it is possible to explain up to 90% of body muscle and fat amount variation. With advances in CT, it is possible to perform spiral scanning of the entire body and, in this way, obtain 3D images. The number of slice images depends on the size of the object and the thickness of the cut, which can be 0.6 mm, the most common being between 2 and 5 mm [33]. The slice thickness and their number are factors that affect CT accuracy. It was found that the slice thickness between 1 mm and 5 mm does not seem to have a substantial impact on the accuracy of the prediction of body or carcass composition, but it does have a direct effect on the time acquisition of CT images. Thicker slices lead to a faster acquisition time, but slighter slice thickness adds further information to CT images [45]. In a work with goats it was possible to scan the entire carcass in about 60 s [44].

Accurate information about phenotypic characteristics is fundamental to genetic progress for meat production, and CT can meet this challenge [47]. Furthermore, with the steady progress in the field of image analysis, it is possible to obtain information based on 3D images quickly and accurately, which reinforces the ability of CT to obtain information both in vivo and on the carcass [35,48,49]. As mentioned previously, most of the CT work is focused on predicting carcass composition. However, there are other studies aimed to predict internal fat deposits [42,46], spine length [50] and muscularity [51]. All these studies clearly showed that CT is competent to evaluate those features. The results from these studies have implications for meat production, as it will be possible to monitor fat reserves throughout the production cycle [42,46], increase the number and length of the lumbar vertebrae and support the muscularity concept improving the conformation and leanness of the carcass [51,52]. CT has also been used to predict meat quality indicators. Table 3 shows the results for the prediction of percentage intramuscular fat (IMF), shear force and some sensory attributes of the *Longissimus thoracis et lumborum* (LM) muscle.

The results for estimating IMF are quite variable (R^2^ between 0.36 and 0.70). Concerning shear force and the sensory attributes, CT does not show the ability to explain its variation with R^2^ ranging between 0.02 and 0.13 [45,55]. The results found for the IMF open the possibility of monitoring the animals for this characteristic, although this requires attention because of the inverse relationship between carcass fat and lean yield [56]. The reduced scanning time of the new equipment and the possibility of making a simultaneous analysis of several small objects allows the optimization of the use of CT machines for cuts or individual muscles [33,55]. The results found with sheep and goats confirm that CT is one of the most relevant tools to obtain accurate phenotypic information which allows the measures to be included either in a breeding program or as a sorting criteria in a meat processor, which will allow better meat quality and potential valorization by the market [14].

### 2.3. Magnetic Ressonace Imaging

Magnetic resonance imaging (MRI) is pointed out as the most accurate technique for determining the body composition and carcass composition of meat-producing animals [14]. This technique has several attributes that are not observed in the other image techniques previously described. MRI allows the provision of complete contrast among or within the various tissues and organs in the lean-tissue category, which makes it possible to measure volumes very precisely [57,58].

MRI is widely available for clinical purposes in humans, but its application in animals, and particularly to meat science, is limited, which is undoubtedly linked to the significant constraints that this technique presents. These constraints are related to the high cost, the extended scanning time, the high operating costs, including temperature control and general maintenance, and the need for a Faraday cage around the device [58]. A directory of MRI facilities available in 2015 in Europe [33] shows that only three institutions conducting MRI research in meat species (Institut National de Recherche en Sciences et Technologies pour Environnement et Agriculture—IRSTEA, Rennes, France; Livestock Center of the Ludwig—LMU, Munich, Germany, and Institute of Diagnostic Imaging and Radiation Oncology, Kaposvár, Hungary). The MRI has been used in poultry, turkey, fish, sheep and, most extensively, swine [33]. For goats, no work with MRI was identified after consulting the Web of Science. Given MRI’s precision, this technique is considered a reliable alternative to dissection [59] or can be seen as the reference method for other techniques [58].

For sheep, the results show that MRI has the potential to predict the body composition and carcass traits accurately [60,61,62,63,64]. Table 4 presents studies to predict in vivo the amount and percentage of body muscle and fat from MRI in sheep. The results show a highly accurate prediction of body muscle and fat [60,61]. The MRI proved to have the potential to be a reference technique for body and phenotypic assessment of carcass traits [65] but the limitations linked to its cost prevent it from being used more widely. In any case, the development of equipment [66] and algorithms for image analysis [67] will continue to position this technique as an option for animal and meat science.

## 3. Spectroscopic Techniques for Assessment of Carcass and Meat Quality of Sheep and Goats

Sheep and goat meat and meat products need monitoring to ensure their quality and safety. In recent years, the meat industry has been investing in cutting-edge technology to do this monitoring, which requires fast, non-destructive and easy-to-use technology. Optical technologies are gaining importance, and among them, the ones that have deserved more attention are visible and near-infrared reflectance spectroscopy (VIS–NIRS and NIRS), hyperspectral imaging (HSI) and Raman spectroscopy (Raman). This section analyses these optical technologies to assess the quality attributes of sheep and goat meat and meat products.

### 3.1. Visible and Near-Infrared Reflectance Spectroscopy

Near-infrared spectroscopy (NIRS) underwent significant advances in the last decade which allowed it to be applied in animal and meat science playing an essential role in evaluating the quality and safety attributes of the meat [11]. An area of large interaction of NIRS is its potential application in a slaughter line or in the meat processing industry to assess meat quality to guarantee the quality and authenticity of the meat products [68,69]. The NIRS which measures the absorption of electromagnetic radiation in the near-infrared spectrum (750–2500 nm), is fast, reliable, does not use reagents and can be used to control processes in the industry [11,69]. However, the reflectance in the visible regions of the spectrum (350–750 nm) is also pointed out as a promising predictor of the quality of meat [70,71]. Thus, the possibility of combining the visible and near-infrared spectra (350–2500 nm) attracted the attention of the meat industry stakeholders to create a new technology called visible-near infrared spectroscopy (VIS–NIRS). For sheep, the VIS–NIRS has been used for the evaluation of meat attributes in a variety of quantitative applications [72,73]. Table 5 summarizes the research using NIRS and VIS–NIRS to assess meat quality of sheep and goats. After a search in the Web of Science database, only one work dealing goats meat with NIRS was found [74]. Generally, the work on NIRS and VIS–NIRS is centred on static equipment under laboratory conditions. This stance is understandable since it was only recently that research on the application of NIRS and VIS–NIR, online or in real-time, was carried out with the aim to measure, control or predict the quality of sheep meat and carcasses [72]. Working in a slaughter line environment requires specialized equipment that must be versatile, quick and simple to use [75]. In general, the results show a good prediction ability for NIRS and VIS–NIRS as indicated by high R^2^ for different meat attributes [72,74,76,77]. Besides, VIS–NIRS also estimate taste traits but with modest capacity R^2^ < 0.4 [78]. The VIS–NIRS have also been shown to be of interest for discriminating carcasses of lambs originating in pasture-fed and concentrate-fed diets [73]. This work shows that it is possible based on perirenal fat to discriminate between 98.5 and 100% of lamb carcasses accurately.

One aspect that deserves concerns is the portability of equipment. There is a clear need for smaller, hand-held equipment for rapid and real-time classification of lamb carcasses [75]. A recent study [72] tested a NIRS nano instrument that has the potential to be a fast, ultra-compact and cost-effective for predicting IMF lamb meat. Other works have also used hand-held NIRS and VIS–NIRS equipment with positive results and with a very favourable appreciation for the ease with which topside of a hanging lamb carcass is assessed in-situ [75]. The NIRS and VIS–NIRS technologies are currently highly versatile tools used in diverse fields including the food industry and, particularly, in animal science to predict the chemical and physical composition of meat of sheep and goats. Additionally, those techniques are reliable tools for identification and authentication of meat products, which is particularly relevant to ensure PDO and PGI brands of origin.

### 3.2. Hyperspectral Imaging

Hyperspectral imaging (HSI) technology was initially developed for military purposes [81]. Like many other technologies that have developed in the military field, the use of HSI has been extended to several fields of animal science, with enormous success [82]. HSI has assumed a significant role in meat science as a reliable, non-destructive and non-invasive tool in the evaluation of quality and safety of meat and meat products [68,83]. Its characteristics are in line with the need to predict the quality and the classification of meat and, as such, making HSI a very attractive technique for meat quality and safety programs [84,85].

In the past five years, numerous studies have recognized the merits of applying HSI in the meat industry, as it provides a versatile, rapid, non-invasive measurement, without complicated and tedious sample preparation, reagents or procedures [85]. In this section, emphasis will be placed on the use of HSI to evaluate attributes in sheep meat to evaluate sensory, chemical, and technological and classification attributes. Additionally, the classification proposed by [85] for the quality attributes of red meats will be used. For goats, like other techniques, there is no information.

Table 6 shows sensory, chemical, and technological, adulteration, authentication and discrimination attributes, which were investigated using HSI for sheep meat.

Sensory attributes significantly influence consumers’ assessment of meat quality. Generally, colour is used as an indicator of meat freshness, and an attractive and stable colour in meat is what the consumer values [86]. In general, HSI predicts meat colour very well [87]. Several studies used HSI as a technique for measuring meat colour, and it was found that HSI and multivariable models have good capacity to predict the colour component L* (R^2^ from 0.77 to 0.97). However, for the other colour components, the predictions show some inconsistencies (R^2^ of 0.48 and 0.84, and R^2^ of 0.26 and 0.82, for b*and a*, respectively). These results point to the need for refinements of the technique in future research [88]. The HSI was used to predict tenderness, and Warner–Bratzler shear force (WBSF) and the result was favourable for WBSF (R^2^ = 0.84) and also for sensory tenderness (R^2^ = 0.69) [89]. Regarding the estimation of the chemical composition by HSI, several studies were carried out with sheep meat [90,91]. In these studies, the results show that HSI allowed estimates with good accuracy for fat (R^2^ > 0.91), protein (R^2^ > 0.80) and water (R^2^ > 0.88). In a more recent study [92], an HSI system (400–1000 nm) was able to estimate the water content of sheep meat, beef and pork, with high accuracy (R^2^ = 0.97). These results confirm that HSI is a technology capable of estimating the chemical composition of red meat.

For lamb, few studies have been conducted using HSI to estimate IMF% and fatty acid content. A recent study, [93] used an HSI system (550–1700 nm) for the simultaneous prediction of the IMF% and the level of 34 fatty acids in LM lamb muscle samples and the results show a modest capacity of HSI to predict the fatty acid content, with R^2^ values between 0.03 for lignoceric acid (C24: 0) and 0.70 for oleic acid (C18: 1c9). Additionally, it was observed that the accuracy for the prediction of IMF%, saturated fatty acid—SFA, monounsaturated fatty acid—MUFA and polyunsaturated fatty acid—PUFA was 0.67, 0.68, 0.70 and 0.53, respectively. The results were pointed out by the authors as promising and, thus, it is possible to anticipate that, with refinement, it will be possible to improve the robustness of HSI to carry out an objective, rapid and non-invasive assessment of the quality of lamb meat. In addition to sensory and chemical attributes, the ability of HSI to predict technological attributes, such as pH, shear force and water holding capacity (WHC), in sheep meat was also investigated. The results are variable, but the HSI shows a capacity to predict those attributes. For example, for the estimation of pH, the models found showed a consistent performance of prediction (R^2^ between 0.38 and 0.71). The HSI was also used to predict the cutting force value of lamb meat obtained either by WBSF or with the MIRINZ technique [88,89].

The results obtained indicate a good accuracy of HSI to predict WBSF (R^2^cv of 0.84 and 0.89), but with lower results with the MIRINZ technique (R^2^ = 0.41). Regarding the WHC a HSI in a system using 237 wavelengths and a PLSR model, made it possible to find an R^2^ value of 0.77 [94]. In a more recent study, the same team tested with good results (R^2^ = 0.92) an HSI system in a project for online monitoring of WHC in beef, lamb and pig [95].

The adulteration of meat is a very challenging domain that has yet to be fully resolved. However, HSI have been used as a reliable solution to identify meat adulteration problems of different species [104,105,106,107]. An HSI system was used to detect the level of adulteration of lamb meat with minced pork, lung, heart and kidney by 2–40%, in 2% increments [98]. The results showed that with a visual evaluation in RGB images, it was not possible to identify the degree of adulteration in the different samples. In contrast, with HSI, this adulteration is clearly distinguished in images. In addition to adulteration with the meat of other species, HSI is also able to detect adulteration of meat, considering its state of conservation (frozen, thawed, fresh and packaged/unpacked). In a similar work, it was found that HSI allowed overall discrimination of 94%, of lamb, beef, or pork meat regardless of their state of conservation (fresh, frozen, thawed, and packing and unpacking) [99].

In lamb carcasses, it was also possible to verify that HSI can discriminate between different muscles and meat of different species. HSI and multivariate analysis [100] in lamb carcasses were able to correctly discriminate 96.7% of four muscles (LM, PM, ST, SM). Other authors [101] show that it is possible to accurately discriminate three muscles (LM, PM, ST) of the lamb carcass.

### 3.3. Raman Spectroscopy

Among the various spectroscopic technologies that have been applied to assess meat quality, Raman spectroscopy (Raman) has shown to be one of those that, in recent years, has received more attention [108,109]. Raman is considered to be of great interest in assessing the composition and quality of meat [108,110,111]. In this section, recent works investigating the use of Raman in the evaluation of meat quality in sheep and goats will be addressed. Raman applied in the evaluation of meat characteristics have been mainly oriented towards pork [109,112] and beef [113,114]. However, there are also several studies in which Raman has been applied to sheep. Table 7 presents results from the application of Raman to sheep meat. For goats, however, little information is available, and only two articles were identified in Web of Science [115,116]. In the first of those studies, aspects related to the origin of the meat were studied and in the second, the characterization of fats from several species, including the goats, was carried out. The Raman application in sheep aimed to predict some meat quality attributes, such as shear force, colour, cooking losses and pH [108]. The prediction of those attributes in lamb LM muscle using Raman is significant, but there is some discrepancy in the results. Examples are the works presented by [117] and [118]. In the first study, the prediction of the shear force of the lamb LM muscle showed reduced accuracy (R^2^cv = 0.06) in contrast to [118], which obtained coefficients of determination (R^2^) of 0.79 and 0.86 for this attribute, measured in two sites of the LM muscle. Good results were also observed for prediction of shear force in the Semimembranosus (SM) muscle [119]. This study reports that the use of Raman is better to predict shear force than traditional predictors such as sarcomere length, pH and particle size. With Raman models, reductions in RMSE of 12.9% and 7.6% were observed in the shear force with SM muscle ageing for one day and five days, respectively. All the works presented in Table 7 were performed with the same portable Raman device and analysed the same muscles (LM and SM) of lambs with similar weight; however, they differ in some aspects of the methodology that may explain the differences found. The state in which the samples were analysed was different. In the work of [117] in measurements made with Raman, fresh muscle samples were used, whereas in the work of [118] Raman measurements were performed after the muscle was frozen and thawed. To understand this issue, [120] carried out a study in which they used Raman to estimate meat quality characteristics in two experiments; one with fresh SM muscle samples and the other with freeze/thaw SM muscle samples. With this work, it was concluded that Raman was not able to predict the shear force after freezing and thawing. However, for other meat quality attributes, the prediction was possible (R^2^ from 0.22 to 0.59).

Several works with Raman have also shown that this technique is capable of classifying samples of meat from different species. For example, [122] applied a combination of Raman with multivariate analysis methods, and neural networks achieving an accuracy between 96.7% and 99.6% in the classification of chicken, bovine, lamb and pork fat. This capacity for discrimination is also observed by others, working with Raman and using principal component analysis, to successfully classify fat samples from seven different types of meat (cattle, sheep, pigs, fish, birds, goats and buffaloes) and their salami products [115]. These authors suggest Raman as a useful tool in the detection of adulterations in the meat industry which will contribute to tempering consumers concerns about the meat they consume.

In addition to this ability to classify quality attributes of meat and fat, Raman was also adequate to predict the concentration of the main groups of fatty acids such as PUFA, MUFA and SFA and also the IMF [121]. The use of Raman to estimate fatty acids has a substantial practical advantage since it goes beyond the time-consuming and tedious processes of extracting and purifying fatty acids. Additionally, traditional methods are expensive, destructive, require chemicals and extensive sample preparation. Raman was also considered valid to discriminate between the SM muscle of fresh tough and tender lamb, using the intensity of spectral peaks that correspond to the tyrosine doublet at 826 and 853 cm^−1^ and α-helix at 930 cm^−1^ [119].

In the development of spectroscopic equipment to be used in the assessment of carcass composition and meat quality, special attention has been paid to its portability and ease of use [111,123]. This attention has been applied to Raman, which rarely requires sample preparation; it is possible to perform an analysis in a few seconds, and there is portable equipment suitable for practical application in the meat industry [108,118]. Raman used in most of the described lamb studies; it is portable equipment that had a robust waterproof encase to protect the sensor [124] and is considered a more versatile approach for application in the meat industry than a bench instrument [108,125]. As with all other spectroscopic techniques, Raman spectra have numerous dependent variables; therefore, it is necessary to use multivariate analysis techniques. The discriminant analysis of partial least squares performed well for classification [122] and the PLSR analysis has been the most used multivariate analysis method for this technique [108].

When compared with other spectroscopic techniques such as NIRS or HSI, relatively few studies have been carried out with Raman to predict meat quality and also to classify meat in sheep and goat species. Of the few studies with sheep, the inconsistent results in some of them did not adequately demonstrate the ability of the Raman technique to assess the quality of sheep meat. Still, the encouraging results and the characteristics of robustness, manoeuvrability and speed analysis are attributes that could elect this technique with great potential for practical use in an industrial meat environment. However, with the advancement of technology, more research will be needed, before Raman can be adopted extensively in the industry.

## 4. Summary of Attributes across All Technologies Discussed

Table 8 summarizes several attributes of the imaging and spectroscopic techniques that were previously presented. The scores given to the different attributes were obtained from the information presented by the various authors. In all techniques, there are significant variations for all the attributes considered, but what is shown to be the most variable are speed and cost. For example, the speed of use depends mostly on the characteristics of the equipment but also on the size of the object. For the cost of equipment, there is also much variation. For example, a CT scanner cost can vary between 80,000 to more than 300,000 €, and an HSI system can vary between 17,000 and 70,000 € [33,126]. The scores were therefore considered for the more common use for each of the techniques. Pictures and information about the imaging and spectroscopic techniques can be found in several works based on the Farm Animal Imaging COST Action FA1102 project [19,33,125,127].

## 5. Conclusions

Meat production and, in particular, ruminant meat production are facing pressures to deliver an environmentally sustainable product with high nutritional value, safe and that can meet the expectations of consumers as well as all stakeholders in the production chain. At the moment, the best possibility of predicting carcass and meat quality seems to be accomplished by combining the traditional classification system with additional measurements provided by imaging and spectroscopic techniques. There are few information/research results available for goats, but since the techniques discussed allow measuring carcass and meat traits well in sheep, it is reasonable to presume that the same could be possible in goats. The application of these techniques to evaluate the quality of carcass and meat of sheep and goats still has much to be learned. However, the results presented in this work for all techniques show that the journey is already underway and, with future technological developments, tangible cost-effective benefits for the industry can be expected through their use.

## Figures and Tables

**Table 1:** Summary of the principal studies to predict fat (% and g) and lean (% and g) of carcass and in vivo of sheep using DXA.

Scanning Object	n	Scanner	Scanning Time	Data Analysis	Composition Trait								Reference
					Fat (%)		Fat (g)		Muscle (%)		Muscle (g)		
					R^2^	RMSE	R^2^	RMSE	R^2^	RMSE	R^2^	RMSE	
Carcass	155	Lunar iDXA	∼3–7 min	SMLR	0.920	0.74	0.920	260	0.780	1.99	0.920	220	[22]
Carcass	155	Lunar iDXA	∼3–7 min	PLSR					0.880	2.68			
Carcass	454	140 kV GADOX	Chain speed	PLSR	0.910	1.19			0.740	1.54			[18] #
Carcass	607	140 kV GADOX	Chain speed	PLSR	0.890	1.32			0.690	1.69			[16] #
Cold carcass				LR	0.750		0.490						[23]
Carcass				LR	0.470		0.130						
Cold carcass	24	140 kV GADOX			0.770	2.48			0.660	2.35			[24] #
Carcass	24	140 kV GADOX			0.700	2.77			0.620	2.43			
Carcass	93	GE Lunar DPX-IQ			0.730	0.90	0.830	177	0.570	1.76	0.880	197	[19]
In vivo	93				0.510	2.22	0.710	229	0.500	1.88	0.570	369	
Carcass	28	Hologic QDR4500A					0.992				0.984		[17]
In vivo	28	Hologic QDR4500A									0.988		
In vivo	59	GE Lunar DPX IQ		SMLR	0.590	2.13	0.670	251	0.490	1.96	0.670	320	[25]
Carcass	59	GE Lunar DPX IQ		SMLR	0.720	1.77	0.820	185	0.520	1.92	0.840	223	
Carcass	50	Norland XR-26	∼2 min	LR			0.860	420			0.900	674	[26]
In vivo	50	Norland XR-26	∼2 min	LR			0.700	710			0.720	1005	
Carcass	60	Hologic QDR4500W		LR	0.942		0.988		0.937		0.985		[21]
Carcass	140	Lunar DPX-L		LR	0.771	2.5					0.930	226	[27]
Frozen carcass	24	Hologic QDR 4500A		LR	0.920	1.2	0.970	163			0.980	232	[28]

n: number of animals or carcasses; SMLR: stepwise multiple linear regression; PLSR: partial least squares regression; LR: linear regression; R^2^: coefficient of determination; RMSE: root mean square error; # percentages determined by computed tomography.

**Table 2:** Summary of applications of computed tomography imaging for prediction of carcass composition of sheep and goats.

Specie	Target		n	CT Image	Anatomical Landmarks	Data Analysis	Tissue (kg)						Reference
							Muscle		Fat		Bone		
							R^2^	RMSE	R^2^	RMSE	R^2^	RMSE	
Sheep	In vivo		21	2D	TV7, LV2, LV5, FEM	LR	0.94	0.508	0.93	0.406	0.73	0.262	[38]
	In vivo	Leg	47	2D	CAV3, CAV4, SV4	LR	0.93		0.95		0.83		[39]
		Shoulder	32		TV6, CV7		0.93		0.96		0.72		
		Mid-Region	104		LV4, TV8		0.89		0.98		0.69		
	In vivo		160	2D	ISC, LV5, TV8		0.92	0.078	0.98	0.097	0.83	0.107	[40]
	Carcass		120			PLSR	0.94	0.710	0.92	0.600			[41]
	In vivo		22	SS (1 mm)		LR	0.94						[42] #
				SS (5 mm)			0.90						
Goats	Carcass		19	SS (5 mm)		LR	0.95		0.65				[43]
	Carcass		10	SS (5 mm)		LR	0.95		0.74		0.47		[44]
	In vivo		20	SS (3 mm)	Volume fatty tissues	LR			0.92	0.760			[36] #

n: number of animals or carcasses; SS: Spiral scanning (slice thickness mm); 2D: two-dimensional cross-sectional scans; anatomical landmarks-TV7: 7th thoracic vertebra; LV2: 2nd lumbar vertebra; LV5: 5th lumbar vertebra; FEM: mid-shaft of the femur; CAV3: 3rd caudal vertebra; SV4: 4th sacral vertebra; TV6: 6th thoracic vertebra; CV7: 7th cervical; LV4: 4th lumbar vertebra; TV8: 8th thoracic vertebra; ISC: ischium; PLSR: partial least squares regression; LR: linear regression; R^2^: coefficient of determination; RMSE: root mean square error; CT: computed tomography; # Body fat.

**Table 3:** Summary of applications of computed tomography imaging for prediction of meat quality attributes of sheep.

Target	Traits	n	CT Image	Anatomical Landmarks	R^2^	RMSE	Reference
In vivo	IMF, %	160	2D	ISC, LV5,LV2,TV8,TV6	0.57	0.608	[53]
In vivo		370	2D	ISC, LV5, TV8	0.51–0.68	0.39–0.48	[54]
		370	2D	LV5	0.51–0.65	0.40–0.48	
Loin	IMF, %	303	SS (8 mm)		0.36	0.620	[55]
In vivo		377	SS (8 mm)		0.51–0.70	0.48–0.38	[45]
		377	SS + 2D	ISC, TV8	0.50–0.71	0.47–0.37	
Loin	Shear force, kgF	303	SS (8 mm)		0.03	−0.830	[55]
In vivo		377	SS (8 mm)		0.02–0.06	0.16–0.16	[45]
		377	SS (8 mm)	ISC, TV8	0.03–0.13	0.16–0.15	
Loin	Texture	303	SS (8 mm)		0.08	−0.530	[55]
	Flavour	303	SS (8 mm)		0.09	−0.370	
	Juiciness	303	SS (8 mm)		0.06	−0.370	
	Liking	303	SS (8 mm)		0.10	−0.390	

n: number of animals or cut; IMF: Intramuscular fat; SS: Spiral scanning (slice thickness mm); 2D: two-dimensional cross-sectional scans; anatomical landmarks-TV7: 7th thoracic vertebra; LV2: 2nd lumbar vertebra; LV5: 5th lumbar vertebra; FEM: mid-shaft of the femur; CAV3: 3rd caudal vertebra; SV4: 4th sacral vertebra; TV6: 6th thoracic vertebra; CV7: 7th cervical; LV4: 4th lumbar vertebra; TV8: 8th thoracic vertebra; ISC: ischium; PLSR: partial least squares regression; LR: linear regression; R^2^: coefficient of determination; RMSE: root mean square error; CT: computed tomography.

**Table 4:** Values of coefficient of determination and residual mean square error of regression models to predict in vivo the amount and percentage of body muscle and fat from MRI in sheep.

LW (kg)	n	Body Tissue								Reference
		Muscle (g)		Fat (g)		Muscle (%)		Fat (%)		
		R^2^	RMSE	R^2^	RMSE	R^2^	RMSE	R^2^	RMSE	
43	31	0.89	243	0.83	183	0.41	1.72	0.67	1.73	[61]
<30	49	0.96	160	0.96	84	0.78	1.57	0.86	1.49	[62]
>30	84	0.91	261	0.94	195	0.91	1.60	0.90	1.64	

n: number of animals; LW: live weight; M: muscle; R^2^: coefficient of determination; RMSE: residual mean square error.

**Table 5:** Summary of the research using visible-near infrared spectroscopy (VIS–NIRS) and near infrared spectroscopy (NIRS) to assess meat quality of sheep and goats.

Technology	Spectrophotometer	Portability	WR (nm)	n	Attributes	Object	Data Analysis	DA (%)	R^2^	RMSE	Reference
VIS–NIRS	Labspec5000	Portable	350–2500	498	IMF	Freeze-dried ground lamb meat	PLSR		0.76	0.41	[72]
VIS–NIRS	Labspec4	Benchtop	350–2500	498	IMF				0.79	0.38	
VIS–NIRS	Trek	Hand-held	350–2500	498	IMF				0.73	0.34	
NIRS	NIRScan Nano Tellspec	Hand-held (Mini)	900–1700	498	IMF				0.27	1.28	
VIS–NIRS	NIRSystems 6500	Benchtop	400–2500	69	Perirenal fat	Pasture #; carcass	PLS-DA	98.6			[73]
				55	Perirenal fat	Indoors ##; carcass		100			
				65	Perirenal fat	Indoors 28 ###; carcass		98.5			
Visible	MINOLTA CM-700d	Portable	400–700	69	Perirenal fat	Pasture #; carcass		98.6			
				55	Perirenal fat	Indoors ##; carcass		94.5			
				65	Perirenal fat	Indoors 28 ###; carcass		92.3			
NIRS	TerraSpec Halo^®^	Hand-held	350–2500	75	IMF	Topside; carcass	PLSR		0.58	0.85	[75]
					IMF	Loin; carcass			0.50	0.91	
VIS–NIRS	NIRSystems 6500	Benchtop	400–2498	232	Taste traits$	LM	PLSR		<0.40		[78]
					IMF				0.84		
					Moisture				0.67		
VIS–NIRS	NIRSystems 6500	Benchtop	400–2500	76	SFA, MUFA, PUFA, CLA	Raw meat			0.41–0.52		[76]
NIRS			700–2500		SFA, PUFA	Ground meat			0.89–0.98		
VIS–NIRS			400–2500		MUFA, CLA				0.84–0.98		
VIS–NIRS	ASD Labspec	Hand-held	500–2000		pH	LM;ST	PLSR		0.49, 0.70		[79]
					Shear force	LM carcass			0.34; 0.30		
					IMF	LM carcass			0.55; 0.63		
VIS–NIRS	ASD FieldSpec	Benchtop	350–2500	250	IMF	LM	PLSR		0.69	1.6	[80]
					SFA				0.60	192.21	
					MUFA				0.60	168.72	
					PUFA				0.67	27.86	
NIRS	InfraAlyzer500	Benchtop	1100–2500	131	DM	Freeze-dried LM	PLSR		0.96	0.38	[77]
				118	Protein				1.00	0.92	
				120	Fat				1.00	0.43%	
NIRS	Master™ N500	Benchtop	420–1000	66	Protein	Goats ground meat	PLSR		0.87	0.43	[74]
				62	Moisture				0.94	0.48	
				16	Fat				0.60	0.49	

n: number of samples or carcasses; WR: wave length range; DA: discrimination ability; R^2^: coefficient of determination; RMSE: root mean square error; IMF: Intramuscular fat; PLSR: partial least square regression; PLS-DA: partial least square discriminant analysis; Pasture#: Fattened at pasture; Indoors##: Stall-fattened indoors on commercial concentrate and straw; Indoors 28###: Finished indoors with concentrate and straw for 28 days after pasture-feeding; Taste traits$: Taste panel traits (texture, juiciness, flavour, abnormal flavour and overall liking); LM: Longissimus thoracis et lumborum muscle; ST: semitendinosus muscle; SFA: saturated fatty acids; MUFA: monounsaturated fatty acids; PUFA: polyunsaturated fatty acids; CLA: conjugated linoleic acid; DM: dry matter.

**Table 6:** Summary of applications of HSI for evaluating quality attributes of sheep meat.

Attributes		n	WR (nm)	Data Analysis	DA (%)	R^2^	References
Sensory*	a*	29	400–1000	PLSR		0.84	[96]
	a*	80	400–863	PCA. SVM		0.48	[88]
	b*	29	400–1000	PLSR		0.82	[96]
	b*	80	400–863	PCA. SVM		0.26	[88]
	L*	42	900–1700	PLSR		0.91	[94]
	L*	29	400–1000	PLSR		0.97	[96]
	L*	80	400–863	PCA. SVM		0.77	[88]
	Tenderness	100	900–1700	PLSR		0.69	[89]
Chemical	Protein	81	900–1700	PLSR		0.85	[95]
	Protein	126	1021–1396	MLR		0.80	[91]
	Water	126	900–1700	PLSR		0.88	[90]
	Water	126	1021–1396	MLR		0.91	[91]
	Fat	126	900–1700	PLSR		0.91	[90]
	Fat	126	1021–1396	MLR		0.95	[91]
	IMF%	1020	550–1700	PLSR		0.67	[93]
	SFA	1020	550–1700	PLSR		0.68	[93]
	MUFA	1020	550–1700	PLSR		0.70	[93]
	FPUFA	1020	550–1700	PLSR		0.53	[93]
Technological	pH	42	900–1700	PLSR		0.65	[94]
	pH	80	400–863	PCA. SVM		0.38	[88]
	pH	2406	550–1700	PLSR		0.71	[93]
	MIRINZ SF	80	400–863	PCA. SVM		0.41	[88]
	WBSF	100	900–1700	PLSR. MLR. SPA		0.84	[89]
	WBSF	128	400–1000	PLSR		0.89	[97]
	WHC	42	900–1700	PLSR		0.77	[94]
	WHC	81	400–1000	PLSR. LS-SVM		0.92	[95]
Adulteration	Minced lamb meat	200	900–1700	PCA. PLSR. MLR		0.98	[98]
	Red-meat products	75	548–1701	CNN	94.4		[99]
Discrimination	Raw meat	29	900–1700	PCA. PLS-DA	98.7		[96]
	LM, PM, ST, SM	30	380–1028	PCA. LMS	96.7		[100]
	LM, PM, ST	105	900–1700	PCA. LDA	100.0		[101]
	Raw meat	61	1000–2500	LDA	100.0		[102]
	Raw meat	90	1000–2500	LDA	87.5		[103]

* Sensory and colour attributes; n: number of samples; LS-SVM: least square support vector machine; MLR: multiple linear regression; PCA: principal WR: wave length range; DA: discrimination ability; PLSR: partial least square regression; PCA: principal component analysis; SVM: support vector machine; MLR: multiple linear regression; LS-SVM: least square support vector machine; CNN: convolution neural networks; PLS-DA: partial least square discrimination analysis; LMS: least mean square LDA: linear discriminant analysis; R^2^: determination coefficient; WHC: water holding capability; IMF%: intramuscular fat percentage; SFA: Saturated Fatty Acid; MUFA: Monounsaturated Fatty Acid; PUFA: Polyunsaturated Fatty Acid: WBSF: Warner–Bratzler shear force; MIRINZ SF: MIRINZ shear force; LM: Longissimus thoracis et lumborum muscle; PM: Psoas major; SM: Semimembranosus; ST: Semitendinosus.

**Table 7:** Summary of Raman spectroscopy studies for evaluating quality attributes of lamb meat.

Quality Attributes		n	Muscle	Ageing Time (day)	Multivariable Analysis	R^2^	R^2^CV	RMSE	RMSECV	Reference
Technological properties	Shear force (N)	70	LM	1	PLSR	0.79		0.11	0.31	[118]
	Shear force (N)	70	LM	1		0.86		0.10	0.26	
	Shear force (N)	80	LM	1	PLSR		0.06	13.60		[117]
	Shear force (N)	80	LM	5				10.00		
	Shear force (N)	80	SM	1	PLSR		0.27		11.48	[119]
	Shear force (N)	81	SM	5			0.17		12.20	
	Cooking loss (%)	70	LM	1		0.79		3.20	0.09	[118]
	Cooking loss (%)	70	LM	1		0.83		0.03	0.08	
	Purge loss (%)	80	SM	1			0.42		0.90	[120]
	Purge loss (%)	80	SM	5			0.33		0.94	
	pH24	80	SM	1	PLSR		0.48		0.12	
	pHu	80	SM	1			0.59		0.07	
	L	80	SM	1			0.32		1.96	
	L	80	SM	5			0.22		1.87	
Fatty acids/IMF	PUFA (mg/100 g)	80	LM	1	PLSR	0.93	0.21		46.57	[121]
	MUFA (mg/100 g)	80	LM	1		0.54	0.16		400.30	
	SFA (mg/100 g)	80	LM	1		0.08	0.01		358.72	
	PUFA:SFA	80	LM	1		0.21	0.13		0.06	
	IMF (mg/100 g)	80	LM	1		0.08	0.02		1.12	

n: number of samples; N: Newton; LM: Longissimus thoracis et lumborum muscle; SM: Semimembranosus muscle; PLSR: partial least squares regression; R^2^: coefficient of determination; R^2^cv: coefficient of determination for cross validation; RMSE: root mean square error; RMSECV: root mean square error of validation; PUFA: Polyunsaturated fatty acid; MUFA: Monounsaturated fatty acid; SFA: Saturated fatty acid; IMF: Intramuscular fat.

**Table 8:** Summary of several attributes of the imaging and spectroscopic techniques.

Attributes	Techniques					
	Imaging			Spectroscopic		
	DXA	CT	MRI	NIRS and VIS–NIRS	HSI	Raman
Fundamentals	X-ray attenuation coefficients (R value) of a low and of a high energy X-ray spectral level related with soft tissue and bone mineral	Attenuation of X-rays passing an object is transformed into Hounsfield units which are related to a given tissue	A phenomenon called nuclear magnetic resonance is the basic principle in which an atomic nuclei with an odd number of protons or neutrons or both (e.g., hydrogen nucleus) will absorb and re-emit radio waves when placed in a magnetic field.	Food molecules contain functional groups like C-H, N-H and O-H, which are closely related to bands in spectra. The VIS–NIRS ranges from 350–2500 nm and NIRS which measures the absorption of electromagnetic radiation in the near-infrared spectrum (750 to 2500 nm)	The HSI spectral ranges from circa 200 nm (ultraviolet range) to 2500 nm (NIR range). The HSI spectral bands cover most food analysis applications. HSI combines imaging with spectroscopy, which simultaneously provides physical and geometrical features of an object	Raman spectroscopy is based on the inelastic scattering of light that occurs when a sample is exposed to a high-energy monochromatic beam of light such as a laser, which interacts with the sample molecules
Target object	In vivo; carcass; cuts; meat	In vivo; carcass; cuts; meat	In vivo; carcass; cuts; meat	Carcass; cuts; meat	Cuts; meat	Carcass; cuts; meat
Potential dependent variables	Tissue and chemical composition; bone density	Tissue and chemical composition; volume and texture	Tissue and chemical composition; volume and texture	Chemical composition; technological parameters; classification	Chemical composition; sensorial and technological parameters; classification	Chemical composition; sensorial and technological parameters; classification
Accuracy	****	****	*****	****	****	*****
Speed	***	**	*	*****	***	****
Cost	***	**	*	****	***	****
Portability	**	*	*	*****	***	*****
Ease to use	****	***	**	****	***	****
References	[14,19]	[14,33]	[14,33]	[72,75]	[84]	[82,108]

DXA: dual-energy X-ray absorptiometry; CT: computer tomography; magnetic resonance imaging; NIRS and VIS–NIRS: near-infrared spectroscopy and visible-near infrared spectroscopy; HSI: Hyperspectral imaging; Raman: Raman spectroscopy. Scores: *—Unfavourable; **—Not very favourable; ***—Favourable ****—Very favourable; *****—Highly favourable; Note: the scores attributed to each technique are based on the several articles [14,33,108,125,126] and considerations that the various authors present throughout the works for the considered attributes.

## References

[1] Hopkins D.L., Gardner G.E., Toohey E.S., Maltin C., Craigie C., Bünger L. (2015). Australian view on lamb carcass and meat quality – the role of measurement technologies in the Australian sheep industry. Farm Animal Imaging—A Summary Report.

[2] Fowler S.M., Hoban J.M., Melville G., Pethick D.W., Morris S., Hopkins D.L. (2018). Maintaining the appeal of Australian lamb to the modern consumer. Anim. Prod. Sci..

[3] Aboah J., Lees N. (2020). Consumers use of quality cues for meat purchase: Research trends and future pathways. Meat Sci..

[4] Wu G., Bazer F.W., Lamb G.C., Bazer F.W., Lamb G.C., Wu G. (2020). Introduction: Significance, challenges and strategies of animal production. Animal Agriculture.

[5] Kristensen L., Støier S., Würtz J., Hinrichsen L. (2014). Trends in meat science and technology: The future looks bright, but the journey will be long. Meat Sci..

[6] Teixeira A., Silva S., Rodrigues S., Toldra F. (2019). 2019. Advances and sheep and goat meat products research. Advances in Food and Nutrition Research.

[7] Narsaiah K., Biswas A.K., Mandal P.K., Biswas A.K., Mandal P.K. (2020). Nondestructive methods for carcass and meat quality evaluation. Meat Quality Analysis.

[8] Ho H., Stanisic H.H.F., Gruchet M., Sun J., Hunter P., Maltin C., Craigie C., Bünger L. (2015). Generic software modules for the meat industry. FAIM Farm Animal Imaging—A Summary Report.

[9] Jørgensen T.M., AL-Tam F., Dahl V.A., Maltin C., Craigie C., Bünger L. (2015). Artifact removal in differential phase contrast x-ray computed tomography. FAIM Farm Animal Imaging—A Summary Report.

[10] Ekiz B., Baygul O., Yalcintan H., Ozcan M. (2020). Comparison of the decision tree, artificial neural network and multiple regression methods for prediction of carcass tissues composition of goat kids. Meat Sci..

[11] Chapman J., Elbourne A., Truong V.K., Cozzolino D. (2020). Shining light into meat–a review on the recent advances in in vivo and carcass applications of near infrared spectroscopy. Int. J. Food Sci. Technol..

[12] Shiel F., Persson C., Furness J., Simas V., Pope R., Climstein M., Hing W., Schram B. (2018). Dual energy X-ray absorptiometry positioning protocols in assessing body composition: A systematic review of the literature. J. Sci. Med. Sport.

[13] Marra M., Sammarco R., De Lorenzo A., Iellamo F., Siervo M., Pietrobelli A., Donini L.M., Santarpia L., Cataldi M., Pasanisi F. (2019). Assessment of body composition in health and disease using bioelectrical impedance analysis (BIA) and dual energy X-ray absorptiometry (DXA): A critical overview. Contrast Media Mol. Imaging.

[14] Scholz A.M., Bünger L., Kongsro J., Baulain U., Mitchell A.D. (2015). Non-invasive methods for the determination of body and carcass composition in livestock: Dual energy X-ray absorptiometry, computed tomography, magnetic resonance imaging and ultrasound: Invited review. Animal.

[15] Pomar C., Kipper M., Marcoux M. (2017). Use of dual-energy x-ray absorptiometry in non-ruminant nutrition research. Rev. Bras. Zootec..

[16] Gardner G.E., Starling S., Charnley J., Hocking-Edwards J., Peterse J., Williams A. (2018). Calibration of an on-line dual energy X-ray absorptiometer for estimating carcase composition in lamb at abattoir chain-speed. Meat Sci..

[17] Hunter T.E., Suster D., Dunshea F.R., Cummins L.J., Egan A.R., Leury B.J. (2011). Dual energy X-ray absorptiometry (DXA) can be used to predict live animal and whole carcass composition of sheep. Small Rum. Res..

[18] Connaughton S.L., Williams A., Anderson F., Kelman K.R., Gardner G.E. (2020). Dual energy X-ray absorptiometry precisely and accurately predicts lamb carcass composition at abattoir chain speed across a range of phenotypic and genotypic variables. Animal.

[19] Scholz A.M., Kremer-Rücker P.V., Wenczel R., Pappenberger E., Maltin C., Craigie C., Bünger L. (2013). Body composition in farm animals by dual energy X-ray absorptiometry. Farm Animal Imaging.

[20] Ponnampalam E.N., Kerr M.G., Butler K.L., Cottrell J.J., Dunshea F.R., Jacobs J.L. (2019). Filling the out of season gaps for lamb and hogget production: Diet and genetic influence on carcass yield, carcass composition and retail value of meat. Meat Sci..

[21] Dunshea F.R., Suster D., Eason P.E., Warner R.D., Hopkins D.L., Ponnampalam E.N. (2007). Accuracy of dual energy X-ray absorptiometry (DXA), weight, longissimus lumborum muscle depth and GR fat depth to predict half carcass composition in sheep. Aust. J. Exp. Agric..

[22] Juárez M., López-Campos Ó., Roberts J.C., Prieto N., Larsen I.L., Uttaro B.M.E.R., Dugan M., Cancino-Baier D., Hosford S., Galbraith J. (2018). Exploration of methods for lam carcass yield estimation in Canada. Can. J. Anim. Sci..

[23] Justice S., Britt J., Miller M., Greene M., Davis C., Koch B., Duckett S., Jesch E. (2018). Predictions of Lean Meat Yield in Lambs Using Dexa and Chemical Analyses Proximate. Meat Muscle Biol..

[24] Gardner G.E., Glendenning R., Brumby O., Starling S., Williams A., Maltin C., Craigie C., Bünger L. (2015). The development and calibration of a dual X-ray absorptiometer for estimating carcass composition at abattoir chain-speed. Farm Animal Imaging—A Summary Report.

[25] Scholz A.M., Mitchell A.D., Ullrey D.E., Baer C.K., Pond W.G. (2010). Body composition: Indirect measurement. Encyclopedia of Animal Science.

[26] Pearce K.L., Ferguson M., Gardner G., Smith N., Greef J., Pethick D.W. (2009). Dual X-ray absorptiometry accurately predicts carcass composition from live sheep and chemical composition of live and dead sheep. Meat Sci..

[27] Mercier J., Pomar C., Marcoux M., Goulet F., Theriault M., Castonguay F.W. (2006). The use of dual-energy X-ray absorptiometry to estimate the dissected composition of lamb carcasses. Meat Sci..

[28] Clarke R.D., Kirton A.H., Bartle C.M., Dobbie P.M. (1999). Application of dual-energy x-ray absorptiometry for ovine carcass evaluation. Proc. N. Z. Soc. Anim. Prod..

[29] McGrath T.A., Frank R.A., Schieda N., Blew B., Salameh J.P., Bossuyt P.M., McInnes M.D. (2020). Diagnostic accuracy of dual-energy computed tomography (DECT) to differentiate uric acid from non-uric acid calculi: Systematic review and meta-analysis. Eur. Radiol..

[30] Haleem A., Javaid M. (2019). 3D scanning applications in medical field: A literature-based review. Clin. Epidemiol. Glob. Health.

[31] Jay N., Bekhit A., El-Din A. (2017). CT Scanning and Ultrasound Techniques for In Vivo Assessment in Meat Processing. Advances in Meat Processing Technology.

[32] Bünger L., Macfarlane J.M., Lambe N.R., Conington J., McLean K.A., Moore K., Glasbey C.A., Simm G., Karuppasamy S. (2011). Use of X-ray computed to.mography (CT) in UK sheep production and breeding. CT Scanning—Techniques and Applications.

[33] Daumas G., Donkó T., Maltin C., Bünger L. (2015). Imaging Facilities (CT & MRI) in EU for Measuring Body Composition.

[34] Bunger L., Menezes A.M., McLean K.A., Gordon J., Yates J., Moore K., Lambe N.R., Maltin C., Craigie C., Bünger L. (2015). Selecting terminal sire breed rams for lean meat percentage–effects on their crossbred lambs. Farm Animal Imaging—A Summary Report.

[35] Bünger L., Moore K., McLean K., Kongsro J., Lambe N., Maltin C., Craigie C., Bünger J. (2014). Integrating Computed tomography into commercial sheep breeding in the UK: Cost and value. Farm Animal Imaging III.

[36] Lerch S., De La Torre A., Huau C., Monziols M., Xavier C., Louis L., Cozler Y.L., Faverdin P., Lambertor P., Chery I. (2020). Estimation of dairy goat body composition: A direct calibration and comparison of eight methods. Methods.

[37] Pomar C., van Milgen J., Remus A., Hendriks W.H., Verstegen M.W.A., Babinszky L. (2019). Precision livestock feeding, principle and practice. Poultry and Pig Nutrition. Challenges of the 21st Century.

[38] Young M.J., Nsoso S.J., Logan C.M., Beatson P.R. (1996). Prediction of carcass tissue weight in vivo using live weight, ultrasound or X-ray CT measurements. Proc. N. Z. Soc. Anim. Prod..

[39] Kvame T., Vangen O. (2006). In-vivo composition of carcass regions in lambs of two genetic lines, and selection of CT positions for estimation of each region. Small Rumin. Res..

[40] Macfarlane J.M., Lewis R.M., Emmans G.C., Young M.J., Simm G. (2006). Predicting carcass composition of terminal sire sheep using X-ray computed tomography. Anim. Sci..

[41] Kongsro J., Røe M., Aastveit A.H., Kvaal K., Egelandsdal B. (2008). Virtual dissection of lamb carcasses using computer tomography (CT) and its correlation to manual dissection. J. Food Eng..

[42] Rosenblatt A.J., Scrivani P.V., Boisclair Y.R., Reeves A.P., Ramos-Nieves J.M., Xie Y., Erb H.N. (2017). Evaluation of a semi-automated computer algorithm for measuring total fat and visceral fat content in lambs undergoing in vivo whole body computed tomography. Vet. J..

[43] Silva S.R., Teixeira A., Monteiro A., Guedes C.M., Ginja M., Maltin C., Craigie C., Bünger L. (2015). Using computer tomography to predict composition of light carcass kid goats. Farm Animal Imaging—A Summary Report.

[44] Silva S.R., Gonçalves C., Santos V., Azevedo J.M., Guedes C., Costa H.R., Teixeira A., Ginja M. (2019). Prediction of carcass composition of the bravia goat breed by computerized tomography. Rev. Port. Zootec..

[45] Clelland N., Bunger L., McLean K.A., Knott S., Matthews K.R., Lambe N.R. (2018). Prediction of intramuscular fat content and shear force in Texel lamb loins using combinations of different X-ray computed tomography (CT) scanning techniques. Meat Sci..

[46] Lambe N.R., Young M.J., Brotherstone S., Kvame T., Conington J., Kolstad K., Simm G. (2003). Body composition changes in Scottish Blackface ewes during one annual production cycle. Anim. Sci..

[47] Rojo-Gimeno C., van der Voort M., Niemi J.K., Lauwers L., Kristensen A.R., Wauters E. (2019). Assessment of the value of informatiIon of precision livestock farming: A conceptual framework. NJAS Wagen. J. Life Sci..

[48] Xiberta P., Boada I., Bardera A., Font-i-Furnols M. (2017). A semi-automatic and an automatic segmentation algorithm to remove the internal organs from live pig CT images. Comput. Electron. Agric..

[49] Ho H., Yu H.B., Gangsei L.E., Kongsro J. (2019). A CT-image based pig atlas model and its potential applications in the meat industry. Meat Sci..

[50] Lambe N.R., Donaldson C.L., McLean K.A., Gordon J., Menezes A.M., Clelland N., Bunger L., Maltin C., Craigie C., Bünger L. (2015). Genetic control of CT-based spine traits in elite Texel rams. Farm Animal Imaging—A Summary Report.

[51] Jones H.E., Lewis R.M., Young M.J., Wolf B.T. (2002). The use of X-ray computer tomography for measuring the muscularity of live sheep. Anim. Sci..

[52] Navajas E.A., Lambe N.R., McLean K.A., Glasbey C.A., Fisher A.V., Charteris A.J.L., Bünger L., Simm G. (2007). Accuracy of in vivo muscularity indices measured by computed tomography and their association with carcass quality in lambs. Meat Sci..

[53] Macfarlane J.M., Young M.J., Lewis R.M., Emmans G.C., Simm G. Using X-Ray Computed Tomography to predict intramuscular fat content in terminal sire sheep. Proceedings of the 56th Annual Meeting of the European Association for Animal Production nº11.

[54] Clelland N., Bunger L., McLean K.A., Conington J., Maltin C., Knott S., Lambe N.R. (2014). Prediction of intramuscular fat levels in Texel lamb loins using X-ray computed tomography scanning. Meat Sci..

[55] Lambe N.R., McLean K.A., Gordon J., Evans D., Clelland N., Bünger L. (2017). Prediction of intramuscular fat content using CT scanning of packaged lamb cuts and relationships with meat eating quality. Meat Sci..

[56] Anderson F., Pethick D.W., Gardner G.E. (2014). The correlation of intramuscular fat content between muscles of the lamb carcass and the use of computed tomography to predict intramuscular fat percentage in lambs. Animal.

[57] Doneva M. (2020). Mathematical models for magnetic resonance imaging reconstruction: An overview of the approaches, problems, and future research areas. IEEE Signal Process. Mag..

[58] Weigand A.C., Schweizer H., Knob D.A., Scholz A.M. (2020). Phenotyping of the Visceral Adipose Tissue Using Dual Energy X-Ray Absorptiometry (DXA) and Magnetic Resonance Imaging (MRI) in Pigs. Animals.

[59] Monziols M., Collewet G., Bonneau M., Mariette F., Davenel A., Kouba M. (2006). Quantification of muscle, subcutaneous fat and intermuscular fat in pig carcasses and cuts by magnetic resonance imaging. Meat Sci..

[60] Baulain U., Henning M. (2001). Untersuchungen zur Schlachtkörper-und Fleischqualität mit Hilfe von MR-Tomographie und MR-Spektroskopie. Arch. Anim. Breed..

[61] Kremer-Rücker P., Bernau M., Mendel C., Steiner A., Gruber E., Scholz A. (2016). Eignung der Magnetresonanztomographie zur Schätzung der Schlachtleistung von Merino-Lämmern. Nova Acta Leopold..

[62] Korn S., Baulain U., Arnold M., Brade W. (2005). Application of Magnetic Resonance Imaging and ultrasound to determine carcass quality in lamb. Zuchtungskunde.

[63] Mendel C., Scholz A.M., Kremer P.V., Förster M., Ringdorfer F., Krenn V., Zeiler M., Steiner A., Wagenpfeil M., Götz K.U. (2007). Messmethoden zur Beurteilung des Schlachtkörperwertes beim Lamm im Vergleich. DGfZ Schr..

[64] Streitz E., Baulain U., Kallweit E. (1995). Untersuchungen zur Körperzusammensetzung wachsender Lämmer mit Hilfe der Magnet-Resonanz-Tomographie (MRT). Züchtungskunde.

[65] Kušec G., Scholz A.M., Baulain U., Kušec I.D., Bernau M. (2016). Non-invasive techniques for exact phenotypic assessment of carcass composition and tissue growth in domestic animals. Acta Agric. Slov..

[66] Smith S., Madden A.M. (2016). Body composition and functional assessment of nutritional status in adults: A narrative review of imaging, impedance, strength and functional techniques. J. Hum. Nutr. Diet..

[67] Caballero D. (2020). Radial textures: A new algorithm to analyze meat quality on MRI. Multimed. Tools Appl..

[68] Kutsanedzie F.Y., Guo Z., Chen Q. (2019). Advances in nondestructive methods for meat quality and safety monitoring. Food Rev. Int..

[69] Nolasco-Perez I.M., Rocco L.A., Cruz-Tirado J.P., Pollonio M.A., Barbon S., Barbon A.P.A., Barbin D.F. (2019). Comparison of rapid techniques for classification of ground meat. Biosyst. Eng..

[70] Shackelford S.D., Wheeler T.L., Koohmaraie M. (2004). Development of optimal protocol for visible and near-infrared reflectance spectroscopic evaluation of meat quality. Meat Sci..

[71] Weeranantanaphan J., Downey G., Allen P., Sun D.W. (2011). A review of near infrared spectroscopy in muscle food analysis: 2005–2010. J. Near Infrared Spectrosc..

[72] Dixit Y., Pham H.Q., Realini C.E., Agnew M.P., Craigie C.R., Reis M.M. (2020). Evaluating the performance of a miniaturized NIR spectrophotometer for predicting intramuscular fat in lamb: A comparison with benchtop and hand-held Vis-NIR spectrophotometers. Meat Sci..

[73] Huang Y., Andueza D.D., de Oliveira L., Zawadzki F., Prache S. (2015). Comparison of visible and near infrared reflectance spectroscopy on fat to authenticate dietary history of lambs. Animal.

[74] Teixeira A., Oliveira A., Paulos K., Leite A., Márcia A., Amorim A., Pereira E., Silva S., Rodrigues S. (2015). An approach to predict chemical composition of goat longissimus thoracis et lumborum muscle by near infrared reflectance spectroscopy. Small Rumin. Res..

[75] Fowler S.M., Morris S., Hopkins D.L. (2020). Preliminary investigation for the prediction of intramuscular fat content of lamb in-situ using a hand-held NIR spectroscopic device. Meat Sci..

[76] Guy F., Prache S., Thomas A., Bauchart D., Andueza D. (2011). Prediction of lamb meat fatty acid composition using near-infrared reflectance spectroscopy (NIRS). Food Chem..

[77] Viljoen M., Hoffman L.C., Brand T.S. (2007). Prediction of the chemical composition of mutton with near infrared reflectance spectroscopy. Small Rumin. Res..

[78] Andrés S., Murray I., Navajas E.A., Fisher A.V., Lambe N.R., Bünger L. (2007). Prediction of sensory characteristics of lamb meat samples by near infrared reflectance spectroscopy. Meat Sci..

[79] Knight M.I., Linden N., Ponnampalam E.N., Kerr M.G., Brown W.G., Hopkins D.L., Baud S., Ball A.J., Borggaard C., Wesley I. (2019). Development of VISNIR predictive regression models for ultimate pH, meat tenderness (shear force) and intramuscular fat content of Australian lamb. Meat Sci..

[80] Pullanagari R.R., Yule I.J., Agnew M. (2015). On-line prediction of lamb fatty acid composition by visible near infrared spectroscopy. Meat Sci..

[81] Shimoni M., Haelterman R., Perneel C. (2019). Hypersectral imaging for military and security applications: Combining myriad processing and sensing techniques. IEEE Geosci. Remote Sens. Mag..

[82] Andueza D., Mourot B.P., Hocquette J.F., Mourot J., Toldra F. (2017). Phenotyping of Animals and Their Meat: Applications of Low-Power Ultrasounds, Near-Infrared Spectroscopy, Raman Spectroscopy, and Hyperspectral Imaging. Lawrie’s Meat Science.

[83] Liu Y., Pu H., Sun D.W. (2017). Hyperspectral imaging technique for evaluating food quality and safety during various processes: A review of recent applications. Trends Food Sci. Technol..

[84] Cheng J.H., Nicolai B., Sun D.W. (2017). Hyperspectral imaging with multivariate analysis for technological parameters prediction and classification of muscle foods: A review. Meat Sci..

[85] Xu J.L., Sun D.W., Toldra F., Nollet L.M.L. (2017). Hyperspectral Imaging Technique for Online Monitoring of Meat Quality and Safety. Advanced Technologies for Meat Processing.

[86] Hung Y., Verbeke W. (2018). Sensory attributes shaping consumers’ willingness-to-pay for newly developed processed meat products with natural compounds and a reduced level of nitrite. Food Qual. Prefer..

[87] Farmer L.J., Farrell D.T. (2018). Beef-eating quality: A European journey. Animal.

[88] Qiao T., Ren J., Yang Z., Qing C., Zabalza J., Marshall S. (2015). Visible hyperspectral imaging for lamb quality prediction. TM Tech. Mess..

[89] Kamruzzaman M., ElMasry G., Sun D.W., Allen P. (2013). Non-destructive assessment of instrumental and sensory tenderness of lamb meat using NIR hyperspectral imaging. Food Chem..

[90] Kamruzzaman M., ElMasry G., Sun D.W., Allen P. (2012). Non-destructive prediction and visualization of chemical composition in lamb meat using NIR hyperspectral imaging and multivariate regression. Innov. Food Sci. Emerg. Technol..

[91] Pu H., Sun D.W., Ma J., Liu D., Kamruzzaman M. (2014). Hierarchical variable selection for predicting chemical constituents in lamb meats using hyperspectral imaging. J. Food Eng..

[92] Kamruzzaman M., Selamat J., Iqbal S. (2016). Food adulteration and authenticity. Food Safety.

[93] Craigie C.R., Johnson P.L., Shorten P.R., Charteris A., Maclennan G., Tate M.L., Agnew M.P., Taukin K.R., Stuart A.D., Reis M. (2017). Application of Hyperspectral imaging to predict the pH, intramuscular fatty acid content and composition of lamb M. longissimus lumborum at 24 h post mortem. Meat Sci..

[94] Kamruzzaman M., ElMasry G., Sun D.W., Allen P. (2012). Prediction of some quality attributes of lamb meat using near-infrared hyperspectral imaging and multivariate analysis. Anal. Chim. Acta.

[95] Kamruzzaman M., Makino Y., Oshita S. (2016). Hyperspectral imaging for real-time monitoring of water holding capacity in red meat. LWT—Food Sci. Technol..

[96] Kamruzzaman M., Makino Y., Oshita S. (2016). Online monitoring of red meat color using hyperspectral imaging. Meat Sci..

[97] Wang S.L., Wu L.G., Kang N.B., Li H.Y., Wang J.Y., He X.G. (2016). Study on Tan-lamb mutton tenderness by using the fusion of hyperspectral spectrum and image information. J. Optoelectron. Laser.

[98] Kamruzzaman M., Sun D.W., El Masry G., Allen P. (2013). Fast detection and visualization of minced lamb meat adulteration using NIR hyperspectral imaging and multivariate image analysis. Talanta.

[99] Al-Sarayreh M., Reis M.M., Yan W.Q., Klette R. (2018). Detection of red-meat adulteration by deep spectral–spatial features in hyperspectral images. J. Imaging.

[100] Sanz J.A., Fernandes A.M., Barrenechea E., Silva S., Santos V., Gonçalves N., Patermain D., Juriom A., Melo-Pinto P. (2016). Lamb muscle discrimination using hyperspectral imaging: Comparison of various machine learning algorithms. J. Food Eng..

[101] Kamruzzaman M., ElMasry G., Sun D.W., Allen P. (2011). Application of NIR hyperspectral imaging for discrimination of lamb muscles. J. Food Eng..

[102] Qiao L., Peng Y., Wei W., Li C. (2015). Identification of main meat species based on spectral characteristics. Proceedings of the 2015 ASABE Annual International Meeting.

[103] Qiao L., Peng Y., Chao K., Qin J., Kim M.S., Chao K., Chin B.A. (2016). Rapid discrimination of main red meat species based on near-infrared hyperspectral imaging technology. Proceedings of the SPIE—Sensing for Agricultural and Food Quality and Safety VIII.

[104] Jiang H., Wang W., Zhuang H., Yoon S.C., Yang Y., Zhao X. (2019). Hyperspectral imaging for a rapid detection and visualization of duck meat adulteration in beef. Food Anal. Methods.

[105] Jiang H., Cheng F., Shi M. (2020). Rapid identification and visualization of jowl meat adulteration in pork using hyperspectral imaging. Foods.

[106] Zhao Z., Yu H., Zhang S., Du Y., Sheng Z., Chu Y., Zhang D., Guo L., Deng L. (2020). Visualization accuracy improvem;ent of spectral quantitative analysis for meat adulteration using Gaussian distribution of regression coefficients in hyperspectral imaging. Optik.

[107] Kamruzzaman M., Makino Y., Oshita S. (2015). Non-invasive analytical technology for the detection of contamination, adulteration, and authenticity of meat, poultry, and fish: A review. Anal. Chim. Acta.

[108] Fowler S.M., Schmidt H., Scheier R., Hopkins D.L., Toldra F., Nollet L.M.L. (2018). Raman spectroscopy for predicting meat quality traits. Advanced Technologies for Meat Processing.

[109] Kucha C.T., Liu L., Ngadi M.O. (2018). Non-destructive spectroscopic techniques and multivariate analysis for assessment of fat quality in pork and pork products: A review. Sensors.

[110] Fowler S.M., Hopkins D.L., Torley P.J., Gill H., Blanch E.W. (2019). Investigation of chemical composition of meat using spatially off-set Raman spectroscopy. Analyst.

[111] Beganović A., Hawthorne L.M., Bach K., Huck C.W. (2019). Critical review on the utilization of handheld and portable Raman spectrometry in meat science. Foods.

[112] Andersen P.V., Wold J.P., Gjerlaug-Enger E., Veiseth-Kent E. (2018). Predicting post-mortem meat quality in porcine longissimus lumborum using Raman, near infrared and fluorescence spectroscopy. Meat Sci..

[113] Cama-Moncunill R., Cafferky J., Augier C., Sweeney T., Allen P., Ferragina A., Sullivan C., Cromie A., Hamill R.M. (2020). Prediction of Warner-Bratzler shear force, intramuscular fat, drip-loss and cook-loss in beef via Raman spectroscopy and chemometrics. Meat Sci..

[114] Ostovar Pour S., Fowler S.M., Hopkins D.L., Torley P., Gill H., Blanch E.W. (2020). Differentiating various beef cuts using spatially offset Raman spectroscopy. J. Raman Spectrosc..

[115] Boyaci I.H., Uysal R.S., Temiz T., Shendi E.G., Yadegari R.J., Rishkan M.M., Velioglu H.M., Tamer U., Ozay D.S., Vural H. (2014). A rapid method for determination of the origin of meat and meat products based on the extracted fat spectra by using of Raman spectroscopy and chemometric method. Eur. Food Res. Technol..

[116] Saleem M., Amin A., Irfan M. (2020). Raman spectroscopy based characterization of cow, goat and buffalo fats. J. Food Sci. Technol..

[117] Fowler S.M., Schmidt H., van de Ven R., Wynn P., Hopkins D.L. (2014). Raman spectroscopy compared against traditional predictors of shear force in lamb m. longissimus lumborum. Meat Sci..

[118] Schmidt H., Scheier R., Hopkins D.L. (2013). Preliminary investigation on the relationship of Raman spectra of sheep meat with shear force and cooking loss. Meat Sci..

[119] Fowler S.M., Schmidt H., van de Ven R., Wynn P., Hopkins D.L. (2014). Predicting tenderness of fresh ovine semimembranosus using Raman spectroscopy. Meat Sci..

[120] Fowler S.M., Schmidt H., van de Ven R., Wynn P., Hopkins D.L. (2015). Predicting meat quality traits of ovine m. semimembranosus, both fresh and following freezing and thawing, using a hand held Raman spectroscopic device. Meat Sci..

[121] Fowler S.M., Ponnampalam E.N., Schmidt H., Wynn P., Hopkins D.L. (2015). Prediction of intramuscular fat content and major fatty acid groups of lamb M. longissimus lumborum using Raman spectroscopy. Meat Sci..

[122] Beattie J.R., Bell S.E.J., Borggaard C., Fearon A.M., Moss B.W. (2007). Classification of adipose tissue species using Raman spectroscopy. Lipids.

[123] Santos C.C., Zhao J., Dong X., Lonergan S.M., Huff-Lonergan E., Outhouse A., Carlson K.B., Prusa K.J., Fedler C.A., Yu C. (2018). Predicting aged pork quality using a portable Raman device. Meat Sci..

[124] Schmidt H., Sowoidnich K., Maiwald M., Sumpf B., Kronfeldt H.D., Vo-Dinh T., Lieberman R.A., Gauglitz G. (2009). Handheld Raman sensor head for in-situ characterization of meat quality applying a mircosystem 671nm diode laser. Proceedings of the SPIE—Sensing for Agricultural and Food Quality and Safety VII.

[125] Craigie C.R., Fowler S., Knight M., Stuart A., Hopkins D., Reis M.M., Maltin C., Craigie C., Bünger L. (2015). Spectral imaging techniques for predicting meat quality—An Australasian perspective. FAIM Farm Animal Imaging—A Summary Report.

[126] Feng C.H., Makino Y., Oshita S., Martín J.F.G. (2018). Hyperspectral imaging and multispectral imaging as the novel techniques for detecting defects in raw and processed meat products: Current state-of-the-art research advances. Food Control.

[127] Font-i-Furnols M., Fulladosa E., Prevolnik Povše M., Čandek-Potokar M., Font-i-Furnols M., Čandek-Potokar M., Maltin C., Prevolnik Povše M. (2015). Future trends in non-invasive technologies suitable for quality determinations. A Handbook of Reference Methods for Meat Quality Assessment.

